# Comparing Different Typologies of Physical Activities With a Focus on Motivation

**DOI:** 10.3389/fpsyg.2022.790490

**Published:** 2022-05-13

**Authors:** Rafael Ming Chi Santos Hsu, Fernando Luiz Cardoso, Marco Antonio Corrêa Varella, Edvane Marlene Pires, Jaroslava Varella Valentova

**Affiliations:** ^1^Department of Experimental Psychology, Institute of Psychology, University of São Paulo, São Paulo, Brazil; ^2^Center of Physical Education, Physiotherapy, and Sports, State University of Santa Catarina, Florianópolis, Brazil; ^3^Department of Experimental Psychology, Institute of Psychology, University of São Paulo, São Paulo, Brazil; ^4^Psychological Orientation Nucleus, Police Academy, Civil Police of São Paulo, São Paulo, Brazil

**Keywords:** motivation, classification, psychology, physical activity, movement, health

## Abstract

There are numerous classifications of physical activities (PAs). However, they lack precise grouping criteria and tend to vary according to each author. Among other factors, the individual’s level of motivation is considered an important aspect of PA maintenance. In this study, we aimed to (1) compare several PAs according to intrinsic (Interest, Enjoyment, and Competence) and extrinsic (Appearance, Fitness/Health, and Social) motives and (2) analyze PAs with and without previous grouping to explore which PAs are more similar based on the different motivational subscales. We recruited 1,421 physically active Brazilian participants (mean age = 26.83, SD = 10.49). The participants stated which PA they practiced most frequently, and they answered the revised motivation for PA measure. The data were analyzed with multivariate general linear models and Kruskal–Wallis. We found that some PAs consistently differed from others regarding motivational subscales. For example, participants practicing Walking showed less Interest/Enjoyment and Competence motives than participants engaged in several other PAs. Pilates was highlighted by a particularly low level of Social motivation in comparison to other PAs. Furthermore, using the previously suggested categorization of PAs, we also showed consistent distinctions considering each motivational subscale. Specifically, one group of PA (categorized as more Complex, Team, Vigorous, Hybrid, and Combative) scored higher on intrinsic motivation, while the other group of activities (categorized as more Organized, Individual, Moderate, Strength, and Rhythmic) scored higher on Appearance and Fitness/Health motives. Our results thus provide initial evidence for possible new methods of grouping PA types that can improve maintenance behavior using motivation as a grouping factor.

## Introduction

There is a considerable variety and heterogeneity within the broad category of physical activities (PAs). Researchers and practitioners are still discussing how to better understand and group the possible subgroups of PAs. Several classification systems have been proposed for the purpose of grouping, differentiating, and organizing PAs. PA can be classified by context, such as leisure, commuting, household, and work (Streb et al., 2019). Leisure-time PAs are commonly sub-classified into sports and exercises, and each of them can be further subdivided. For example, sports can be individual or team (e.g., Molanorouzi et al., 2015), while exercises can be classified as resistance, flexibility, endurance, balance, etc. (Caspersen et al., 1985).

The intensity of energy expenditure can also classify PA as light, moderate, or vigorous (Ainsworth et al., 2011). PA can further be divided into aerobic or anaerobic, depending on the metabolic requirements (U.S. Department of Health and Human Services, 1996). In the Compendium of Physical Activities, Ainsworth et al. (2011) used a heading to group types of PAs, each sub-classified according to metabolic equivalents of effort/energy expenditure. For example, sexual activity can be passive, general, or active; dance can be aerobic, ballroom, ballet, etc.; sports can be tennis, badminton, karate, volleyball, etc.; and conditioning exercises can be calisthenics, stretching, resistance training, etc.

Cardoso et al. (2020) proposed a system that classifies PAs as predominantly oriented by either combativeness or rhythmicity. The authors define combativeness as PAs with a high level of physical contact and competition, while rhythmicity in PAs involves music and dance. This classification varies with the sex and sexual orientation of the individual. Heterosexual men and homosexual women were more likely to engage in football, a more combative PA, whereas heterosexual women and homosexual men were reported to be more likely to engage in ballet, a more rhythmic form of PA (Cardoso et al., 2020). Other authors, for example, Davison et al. (2002) and Varella et al. (2017), similarly suggested division into more general PA and artistic/aesthetic PA, such as rhythmic gymnastics, baton twirling, or figure skating, which overlap with the rhythmicity category. Another classification considered in the field of motor learning is Complexity–Organization (Naylor and Briggs, 1963), in which PAs are classified as more or less complex or organized. Organization refers to the demands placed on an individual related to interrelationships between the parts/dimensions of the task, while Complexity refers to the demand placed on an individual related to the processing of information and/or memory storage by each part/dimension of the task independently (Naylor and Briggs, 1963).

However, there is a lack of classification systems for PAs based on basic psychological properties, such as motivation. Motivation is composed of multiple evolved fundamental systems that guide individuals toward survival and reproduction (Kenrick et al., 2010), and thus, categorization of PAs according to individual motives can be valuable because it may naturally organize and anchor the different PAs into universal psychological tendencies.

Motivation is a complex and rich concept that is defined and approached in numerous ways, often contradictory, either in common sense or within specialized areas of scientific disciplines, such as Psychology (Roberts and Nerstad, 2020). In general, motivation modulates and coordinates the direction, vigor, and composition of behavior that arises from a wide variety of internal, environmental, and social sources (Shizgal, 2001). Motivation can be defined as variations in levels of wanting, wishes, and aspirations that are capable of influencing one’s intention and behavior (Ryan and Deci, 2000; Baumeister, 2016; Reeve, 2018).

In the field of sports psychology, two theories are dominant (Roberts and Nerstad, 2020): Achievement Goal Theory and Self-Determination Theory. The Achievement Goal Theory focuses on the goals established to guide decision-making, engagement levels, and responses to achievement outcomes (Roberts and Nerstad, 2020). The Achievement Goal Theory popularized the distinction between motives as predominantly egocentric or task-oriented (Jagacinski and Strickland, 2000). For instance, someone may decide to engage in a particular type of PA with the primary purpose of mastering/learning or of appearing skilled to others. The Self-Determination Theory popularized the distinction between intrinsic (e.g., interest, enjoyment, and competence) and extrinsic motives (e.g., fitness, health, appearance, and socialization) (Ryan and Deci, 2000). For example, someone might be more motivated to follow their interests and inherent abilities or to socialize, thus engaging in team PAs as a result.

In both theoretical and empirical research, studies addressing motivation and participation/choice of PA are mostly oriented by Self-Determination Theory (e.g., Frederick and Ryan, 1993; Ryan et al., 1997; Andrade Bastos et al., 2006; Molanorouzi et al., 2015; Borges et al., 2021; Jenkins et al., 2021; Varella, 2021). For example, Andrade Bastos et al. (2006) found that fitness participants scored higher on appearance and social motives than walking participants. Molanorouzi et al. (2015) found that some PA groups distinguished themselves from all others according to each subscale analyzed (e.g., racquet sports participants had significantly higher motives for mastery and competition compared to the rest of the sample). Ryan et al. (1997) found that aerobics participants reported higher body-related and less competence/enjoyment motives than taekwondo participants. Varella (2021) found that the motivation to apply for a university career in Physical Education (along with Chemistry and Dentistry) as a proxy for a lifetime commitment to sports and exercises is 3.74–8.42 times more intrinsically motivated than it is extrinsically motivated. In a study conducted during a COVID-19 lockdown in New Zealand, Jenkins et al. (2021) found positive associations between doing PA with psychological well-being, with nature-based PAs (i.e., performed “around natural environments”) being stronger predictors of increased intrinsic motivation than non-nature-based PAs. In fact, activities promoting autonomy, competence, and relatedness correlate to higher well-being (Ryan and Deci, 2000), and adherence to PA can be such an example (see Ryan et al., 1997).

In addition to the Achievement Goal Theory and Self-Determination Theory, evolutionary approaches were included to explain PA practice, mainly regarding sex differences in motives for sports (e.g., Deaner et al., 2015; Apostolou and Lambrianou, 2017). In particular, the Renovated Pyramid of Needs based on evolutionary-relevant goals proposes seven fundamental motivational systems: Immediate Physiological Needs, Self-Protection, Affiliation, Status/Esteem, Mate Acquisition, Mate Retention, and Parenting (Kenrick et al., 2010). These systems can be attained through PA.

Previous studies focused on comparing different categories of PAs according to motivational subscales (e.g., Frederick and Ryan, 1993; Molanorouzi et al., 2015; Hsu and Valentova, 2020). Sport participants reported greater intrinsic motives, whereas exercise participants reported more bodily related motives (Frederick and Ryan, 1993; Hsu and Valentova, 2020). Frederick and Ryan (1993) were pioneers in showing the importance of comparing more categories of PA than in previous studies: individual sports, team sports, and exercises (“fitness activities” in the original). We highlight two more recent studies, Molanorouzi et al. (2015) and Hsu and Valentova (2020), that followed similar reasoning and proposed additional categories to group PA. Hsu and Valentova (2020) proposed an update of Frederick and Ryan’s (1993) model by adding a category labeled “Body/Movement Practices,” and their results showed an important distinction between Body/Movement Practices as an intermediate category between sports and exercises, closer to individual sports regarding motivational scores. Molanorouzi et al. (2015) also proposed more categories to group and compare PA: team sports, individual racing plus bowls, racquets, exercises, and martial arts. However, they did not offer any theoretical reasons for their grouping. All the previous categorizations share the limitation of not being data-driven, which can lead to stereotyping or grouping of activities that, in fact, do not create homogenous groups.

We aimed to analyze PAs with and without previous grouping and explore which PAs were more similar according to different motivational subscales. The present research is exploratory, and we hypothesized that some PAs might present a certain difference in grouping compared to others, despite having the same classification system as previous authors. For instance, walking and running are both considered organized exercises but differ considerably depending on the criterion used, such as intensity or energy expenditure. Similarly, rowing and swimming are considered organized exercises, although their participant counts vary significantly; one is usually categorized as a group activity, while the other is classified as an individual activity. As a result, individuals may have disparate scores in certain motives, which may justify their disparate categorization.

## Materials and Methods

### Participants

Participants were recruited using convenience samples from social networks (Facebook and WhatsApp) and mailing lists at one of the most well-ranked Brazilian public universities. We recruited 1,421 participants who stated themselves as physically active (mean age = 26.83, SD = 10.49), 564 men (mean age = 28.05, SD = 11.40), and 856 women (mean age = 25.98, SD = 9.70).

### Instruments

The participants (total *N* = 1,294) indicated their most frequently practiced activity. Table 1 shows all activities included in this study, their respective numbers of participants, and their classification based on previous studies. Since the previous studies have not classified all PAs included in our dataset or did not have precise operational definitions, the first and second authors categorized all PAs together based on the definitions and similar PAs from previous studies. Table 2 shows the total number of PAs split into each classification system. This study is part of a broader project (see Hsu and Valentova, 2020; Hsu, 2022), and here we report the instruments analyzed in this specific study.

**TABLE 1:** Distribution of groupings of PAs.

	Total *N*	%	Level of PA Organization^1^	Number of participants	Physiological Demand^2^	Intensity of energy expenditure^3^	Motor orientation^4^
Strength	344	26.6	Complexity	Individual	Strength	Moderate	Rhythmicity
Running	151	11.7	Organization	Individual	Cardiorespiratory	Vigorous	Rhythmicity
Walking	88	6.8	Organization	Individual	Cardiorespiratory	Light	Rhythmicity
Swimming	69	5.3	Complexity	Individual	Cardiorespiratory	Vigorous	Water Practices
Pilates	63	4.9	Complexity	Individual	Hybrid	Moderate	Rhythmicity
Futsal	45	3.5	Complexity	Team	Hybrid	Vigorous	Combativeness
Soccer	40	3.1	Complexity	Team	Hybrid	Vigorous	Combativeness
Volleyball	47	3.6	Complexity	Team	Hybrid	Vigorous	Combativeness
Handball	33	2.6	Complexity	Team	Hybrid	Vigorous	Combativeness
Basketball	32	2.5	Complexity	Team	Hybrid	Vigorous	Combativeness
Dances	31	2.4	Complexity	Both	Cardiorespiratory	Vigorous	Rhythmicity
Tennis	29	2.2	Complexity	Both	Hybrid	Vigorous	Combativeness
Cycling	27	2.1	Organization	Individual	Cardiorespiratory	Vigorous	Rhythmicity
Athletics	26	2.0	Complexity	Individual	Hybrid	Vigorous	Combativeness
Rugby	19	1.5	Complexity	Team	Hybrid	Vigorous	Combativeness
Yoga	25	1.9	Complexity	Individual	Hybrid	Light	Rhythmicity
Ballet	24	1.9	Complexity	Individual	Cardiorespiratory	Moderate	Rhythmicity
Bycycling/Bike	21	1.6	Organization	Individual	Cardiorespiratory	Moderate	Rhythmicity
Kung Fu	19	1.5	Complexity	Individual	Hybrid	Vigorous	Combativeness
Extreme Conditioning Program	17	1.3	Complexity	Individual	Strength	Vigorous	Rhythmicity
Functional Training	16	1.2	Complexity	Individual	Strength	Vigorous	Rhythmicity
Karate	16	1.2	Complexity	Individual	Hybrid	Vigorous	Combativeness
Gymnastics	14	1.1	Complexity	Individual	Hybrid	Moderate	Rhythmicity
Muay Thai	13	1.0	Complexity	Individual	Hybrid	Vigorous	Combativeness
Table Tennis/Badminton	12	0.9	Complexity	Both	Hybrid	Moderate	Combativeness
Pole Dance	12	0.9	Complexity	Individual	Cardiorespiratory	Moderate	Rhythmicity
Boat (Rowing/Canoeing)	11	0.9	Organization	Both	Hybrid	Vigorous	Water Practices
Aerobics	10	0.8	Organization	Individual	Cardiorespiratory	Vigorous	Rhythmicity
Boxing	9	0.7	Complexity	Individual	Hybrid	Vigorous	Combativeness
Jiu-Jitsu	8	0.6	Complexity	Individual	Hybrid	Vigorous	Combativeness
Judo	8	0.6	Complexity	Individual	Hybrid	Vigorous	Combativeness
Hydrogymnastics	8	0.6	Complexity	Individual	Cardiorespiratory	Moderate	Water Practices
Other Exercises	7	0.5	Complexity	Individual	Hybrid	Moderate	Rhythmicity
Total (sample)	1,294	100.0					

*^1^Based on Naylor and Briggs (1963).*

*^2^Based on Caspersen et al. (1985).*

*^3^Based on Ainsworth et al. (2011).*

*^4^Based on Cardoso et al. (2020).*

**TABLE 2:** Frequencies of groupings of PAs.

	*N*	%
**Level of PA Organization^1^**		100.0
Complexity	898	69.7
Organization	398	30.3
**Number of Participants**		
Individual	988	76.4
Team	216	16.7
Both	83	6.9
**Physiological Demand^2^**		
Strength	375	29.0
Cardiorespiratory	438	33.8
Hybrid	474	37.2
**Intensity of energy expenditure^3^**		
Light	112	8.7
Moderate	501	38.7
Vigorous	674	52,6
**Motor orientation^4^**		
Combativeness	356	27.5
Rhythmicity	843	65.1
Water Practices	88	7.4
Total (sample)	1,294	100.0

*^1^Based on Naylor and Briggs (1963).*

*^2^Based on Caspersen et al. (1985).*

*^3^Based on Ainsworth et al. (2011).*

*^4^Based on Cardoso et al. (2020).*

The participants answered our version of the Motivation for Physical Activity Measure Revised (Ryan et al., 1997) that measures, in 30 items answered on seven-point Likert scales, five motivational subscales related to PA: intrinsic (Competence and Interest/Enjoyment) and extrinsic (Appearance, Fitness/Health, and Social). We translated the instrument from English to Brazilian Portuguese. The internal consistencies measured by Cronbach’s alphas were satisfactory: Interest/Enjoyment (α = 0.92); Competence (α = 0.90); Appearance (α = 0.89); Fitness/Health (α = 0.80), and Social (α = 0.85).

### Procedure

Data were collected online using Qualtrics software (Qualtrics, Provo, UT, United States) at the end of 2016. No reward was provided for participating in the study. The project was approved by the IRB of the Institute of Psychology, University of São Paulo (number 1.506.899, approved on 19 April 2016).

### Statistical Analyses

The analyses were conducted with the Statistical Package for Social Sciences (SPSS) version 21.0.

Regarding the analyses of previous classifications, similar to previous studies (e.g., Frederick and Ryan, 1993), to test for the effect of each classification of PA, we performed separate multivariate general linear models (GLMs) with the type of motivation as the dependent variable, and the types of PA as fixed factors. Tests of Between-Subject Effects were further checked to estimate the main effects, and Estimated Marginal Means with Bonferroni correction were consulted as *post hoc* tests. We also performed correspondence analysis (similarly to Borges et al., 2021) to graphically illustrate the PAs groupings according to each category (Supplementary Figures 1–4).

Regarding the analysis without previously grouping PAs, we performed the Kruskal–Wallis analyses of variance with all PAs categories as fixed factors and each motivational subscale as the dependent variable. Significance was established at 0.05.

## Results

The GLM showed significant effects of Complexity/Organization on motives for PAs [Wilks’ Λ = 0.947, *F*(1,2) = 14.22, *p* < 0.001]. As shown in Table 3, PAs with a greater degree of Complexity over-scored the PAs with a greater degree of Organization in Interest/Enjoyment, Competence, and Social motives, while Organization PAs had higher scores than Complexity in Fitness/Health motivation. No significant differences were found in Appearance motives.

**TABLE 3:** Differences between mean scores (±SD) of motivational dimensions divided into Complexity and Organization.

	Complexity Mean (SD)	Organization Mean (SD)	*F* statistics	Eta-squared
Interest/Enjoyment	5.52 (1.42)	5.19 (1.39)	14.54**	0.011
Competence	4.97 (1.53)	4.44 (1.66)	30.74**	0.023
Appearance	4.82 (1.67)	4.68 (1.48)	2.17	0.002
Fitness/Health	5.77 (1.16)	5.93 (1.05)	5.67*	0.004
Social motivation	2.90 (1.59)	2.43 (1.44)	25.51**	0.019

*p < 0.005; **p < 0.001.

The GLM showed that the number of participants in each activity had a significant effect on motivation for PAs [Wilks’ Λ = 0.674, *F*(2,3) = 55.83, *p* < 0.001]. As shown in Table 4, Individual PAs differed significantly from Team PAs on all motivational subscales, with the intermediate category (Both) showing lower scores compared to the Team only for Social motives. Individual PAs had the highest scores on Fitness/Health and Appearance motives, while Team and Both scored the highest on Interest/Enjoyment and Competence motives.

**TABLE 4:** Differences between mean scores (±SD) of motivational dimensions divided according to predominant Number of Participants.

	Individual Mean (SD)	Team Mean (SD)	Both Mean (SD)	*F* statistics	Eta-squared
Interest/Enjoyment	5.17 (1.48)^2^	6.27 (0.75)^1^	6.15 (0.82)^1^	71.87**	0.101
Competence	4.65 (1.64)^2^	5.40 (1.21)^1^	5.14 (1.42)^1^	22.70**	0.034
Appearance	5.04 (1.54)^1^	3.94 (1.59)^2^	3.84 (1.45)^2^	61.53**	0.087
Fitness/Health	5.98 (1.03)^1^	5.28 (1.29)^2^	5.33 (1.32)^2^	45.06**	0.066
Social motivation	2.36 (1.38)^3^	4.25 (1.32)^1^	2.76 (1.56)^2^	181.44**	0.220

*Means (±SD) with the same superscript numbers do not differ from each other (using Bonferroni’s post hoc comparisons).*

**p < 0.001.

The GLM showed significant effects for predominant Physiological Demand on motives for PAs [Wilks’ Λ = 0.706, *F*(2,3) = 48.75, *p* < 0.001]. As shown in Table 5, the three categories of PA differed among almost all motivational subscales, with the Hybrid showing the highest scores on Interest/Enjoyment, Competence, and Social motives, while Strength showed the highest scores on Fitness/Health and Appearance motives. Cardiorespiratory PAs had an intermediate position in all subscales.

**TABLE 5:** Differences between mean scores (±SD) of motivational dimensions divided according to predominant Physiological Demand.

	Strength Mean (SD)	Cardiorespiratory Mean (SD)	Hybrid Mean (SD)	*F* statistics	Eta-squared
Interest/Enjoyment	4.86 (1.61)^3^	5.36 (1.39)^2^	5.91 (1.08)^1^	62.49**	0.089
Competence	4.54 (1.66)^2^	4.58 (1.66)^2^	5.23 (1.36)^1^	27.35**	0.041
Appearance	5.77 (1.23)^1^	4.61 (1.52)^2^	4.15 (1.61)^3^	130.07**	0.168
Fitness/Health	6.16 (0.94)^1^	5.83 (1.13)^2^	5.54 (1.20)^3^	33.17**	0.049
Social motivation	2.16 (1.25)^3^	2.55 (1.47)^2^	3.41 (1.61)^1^	82.15**	0.113

*Means (±SD) with the same superscript numbers do not differ from each other (using Bonferroni’s post hoc comparisons).*

**p < 0.001.

The GLM showed significant effects of the type of Energy Expenditure on motives for PAs [Wilks’ Λ = 0.750, *F*(2,3) = 39.66, *p* < 0.001]. As shown in Table 6, Vigorous PAs differed from the other types on almost all motivational subscales, scoring the highest scores on Interest/Enjoyment, Competence, and Social motives. Moderate PAs had the highest scores on Appearance and Fitness/Health motives, while Light PAs showed lower scores than Moderate or Vigorous in all motivational subscales.

**TABLE 6:** Differences between mean scores (±SD) of motivational dimensions divided according to predominant Intensity of Energy Expenditure.

	Light Mean (SD)	Moderate Mean (SD)	Vigorous Mean (SD)	*F* statistics	Eta-squared
Interest/Enjoyment	4.79 (1.46)^2^	4.97 (1.56)^2^	5.86 (1.13)^1^	75.98**	0.106
Competence	3.70 (1.69)^3^	4.54 (1.64)^2^	5.18 (1.40)^1^	57.96**	0.083
Appearance	4.15 (1.68)^2^	5.38 (1.46)^1^	4.43 (1.58)^2^	64.71**	0.092
Fitness/Health	5.65 (1.22)^2^	5.99 (1.05)^1^	5.72 (1.16)^2^	9.74**	0.015
Social motivation	2.10 (1.25)^2^	2.19 (1.27)^2^	3.29 (1.61)^1^	94.88**	0.129

*Means (±SD) with the same superscript numbers do not differ from each other (using Bonferroni’s post hoc comparisons).*

**p < 0.001.

The GLM showed significant effects of Motor Orientation on motives for PAs [Wilks’ Λ = 0.708, *F*(2,3) = 48.22, *p* < 0.001]. As shown in Table 7, Combativeness significantly differed from Rhythmicity on all motivational subscales, with Water Practices always showing intermediate values. Rhythmicity had the highest scores on Fitness/Health and Appearance motives, while Combativeness had the highest scores on Interest/Enjoyment, Competence, and Social motives.

**TABLE 7:** Differences between mean scores (±SD) of motivational dimensions divided according to predominant Motor Orientation.

	Combativeness Mean (SD)	Rhythmicity Mean (SD)	Water Mean (SD)	*F* statistics	Eta-squared
Interest/Enjoyment	6.15 (0.85)^1^	5.08 (1.52)^3^	5.74 (1.05)^2^	83.21**	0.115
Competence	5.42 (1.22)^1^	4.52 (1.66)^3^	5.02 (1.45)^1^	43.73**	0.064
Appearance	4.11 (1.63)^3^	5.07 (1.54)^1^	4.64 (1.43)^2^	48.23**	0.070
Fitness/Health	5.47 (1.25)^2^	5.96 (1.06)^1^	5.88 (0.94)^1^	25.16**	0.038
Social motivation	3.86 (1.48)^1^	2.26 (1.32)^3^	3.09 (1.56)^2^	168.75**	0.208

*Means (±SD) with the same superscript numbers do not differ from each other (using Bonferroni’s post hoc comparisons).*

**p < 0.001.

Regarding the effects of using no categories of PA on subscales of motivation, the Kruskal–Wallis test revealed a statistically significant effect of PA on Interest/Enjoyment, χ^2^ (32) = 298.56, *p* < 0.001. Overall, Walking was the least Interest/Enjoyment-motivated PA.

The multiple comparison rank medians showed that Walking had lower scores in Interest/Enjoyment than Swimming, Karate, Kung Fu, Basketball, Rugby, Tennis, Soccer, Dances, Handball, Extreme Conditioning Program, Volleyball, Futsal, Ballet, and Pole Dance (all *p*’s < 0.05). Also, Aerobics had lower scores than Volleyball, Futsal, and Ballet (all *p*’s < 0.007). There were no other significant differences.

Regarding the effects of PA on Competence motivation, the Kruskal–Wallis test revealed a statistically significant effect of PA on Competence, χ^2^ (32) = 194.04, *p* < 0.001. Walking was the least competence-motivated PA.

The multiple comparison rank medians showed that Walking had lower scores in Competence motivation than Swimming, Karate, Kung Fu, Jiu-Jitsu, Basketball, Rugby, Tennis, Soccer, Dances, Handball, Extreme Conditioning Program, Strength, Running, Athletics, Volleyball, Futsal, Ballet, Pilates, and Pole Dance (all *p*’s < 0.033). Also, Aerobics had lower scores than the Extreme Conditioning Program (*p* = 0.021). Bicycling/Bike had lower scores than Kung Fu, Pole Dance, and Extreme Conditioning Program (all *p*’s < 0.038).

Regarding the effects of PA on Social motivation, the Kruskal–Wallis test revealed a statistically significant effect of PA χ^2^ (32) = 375.87, *p* < 0.001. Aerobics and Pilates were the least socially motivated PAs.

The multiple comparison rank medians showed that Aerobics had lower scores than Soccer, Volleyball, Basketball, Futsal, Handball, Athletics, and Rugby (all *p*’s < 0.017). Pilates had lower scores than Extreme Conditioning Program, Swimming, Dances, Tennis, Table Tennis/Badminton, Soccer, Volleyball, Basketball, Futsal, Handball, Athletics, and Rugby (all *p*’s < 0.003).

Regarding the effects of PA on Fitness/Health motivation, the Kruskal–Wallis test revealed a statistically significant effect of PA χ^2^ (32) = 151.97, *p* < 0.001. Handball and Dances were the least Fitness/Health-oriented PAs.

The multiple comparison rank medians showed Handball with lower scores than Running, Swimming, Strength, Functional Training, Jiu-Jitsu, and Extreme Conditioning Program (all *p*’s < 0.039). Dances had lower scores than Strength, Running, and Extreme Conditioning Program (all *p*’s < 0.004).

Regarding the effects of PA on Appearance motivation, the Kruskal–Wallis test revealed a statistically significant effect of PA χ^2^ (32) = 317.40, *p* < 0.001. Yoga, Handball, and Tennis were the least Appearance-oriented PAs.

The multiple comparison rank medians showed that Handball had lower scores than Running, Strength, and Extreme Conditioning Program (all *p*’s < 0.037). Tennis had lower scores than Strength and Running (all *p*’s < 0.038). Yoga had lower scores than Running, Strength, and Extreme Conditioning Program (all *p*’s < 0.025).

## Discussion

We aimed to compare PAs according to five motivation subscales in order to verify possible similarities and differences, either using previously proposed classification systems or a more data-driven analysis.

The results confirm that different classification systems present a similar distinction between extrinsic and intrinsic motives. The Social and intrinsic motives of Interest/Enjoyment and Competence tend to be greater for PAs that are defined as more complex, practiced in teams, involve hybrid physiological predominance, vigorous effort, and a motor orientation of combativeness. On the other hand, extrinsic motives for Fitness/Health and Appearance tend to be greater for PAs that are defined as more organized and individual, with a predominance of strength, moderate effort, and rhythmicity orientation.

According to the analyses without previous grouping, Walking is less motivated by Interest/Enjoyment and Competence than most sports. Furthermore, Aerobics and Pilates were less socially motivated than several sports, mainly collective/team. Appearance motives tend to be lower in Tennis, Yoga, and Handball in comparison to some exercises, such as Strength and Running. Finally, Fitness/Health motives were lower in Dances and Handball in comparison to some exercises. In the same direction, Varella (2021) found that in Dance, the intrinsic motives were 64.31 times higher than the extrinsic motives.

Walking is usually classified as a light to moderate PA (Ainsworth et al., 2011) and can be performed in varied domains with varying intentions (Niven and Markland, 2016). Here, we analyzed Walking as a leisure-time PA. Compared to several other PAs, Walking was one of the least intrinsically motivated. More evidence is needed to test if Walking provides enough opportunities for feelings of competence or pleasure (Morris and Hardman, 1997; Niven and Markland, 2016).

However, the possibility of not being as enjoyable or challenging as other PAs does not negate Walking’s contributions to well-being. In fact, it is known that a Walking intervention can increase exercise self-efficacy and general self-efficacy (Duranso, 2019). Walking is self-regulated, accessible, requires almost no equipment, and has minimal adverse effects (Morris and Hardman, 1997). Thus, Walking is one of the most frequently practiced PAs, especially among starters, which might in part explain the lack of intrinsic motivation. Moreover, some participants may prefer other PAs (such as Tennis) but have fewer or no opportunities to do so. Thus, Walking emerges as a good option, especially for the initiation of PA, since it is a very accessible PA and most people are physically and economically able to do it.

Regarding social motives, we showed that Pilates was the least socially motivated PA. In general, Pilates is a frequently studied PA, and its benefits to health are well-documented (Memmedova, 2018). Despite our results, relatedness is considered an important factor for the maintenance of Pilates, following competence (Lee, 2018). Although social motives may be important in Pilate’s participation, they are not as strong as other motives, for example, competence. In addition, people may prefer other PAs to socialize, such as team sports, whose inherent structure enables them to interact socially and thus fulfill their universal need for relatedness (Baumeister and Leary, 1995; Ryan and Deci, 2000).

Appearance motivation was the strongest for Strength and Running, which were also the most frequently chosen as primary PAs (nStrength = 344, nRunning = 151). Strength leading to muscle hypertrophy and Running leading to fat burning are obviously the most practiced PAs, and appearance is thus among the most important motives for PAs. Appearance motivation is important since it affects everyday lives in many social contexts, including partner selection (Valentova et al., 2021; Davis and Arnocky, 2022). A recent meta-analysis showed that in men, strength/muscularity is the strongest and only consistent predictor of both mating and reproduction (Lidborg et al., 2022). Mating is among the most important basic needs (Kenrick et al., 2010). However, the promotion of highly Appearance-oriented PAs requires caution. For example, Murray et al. (2016) reported relations between appearance orientation with anabolic-androgenic steroid use, eating disorders, and muscle dysphoria. Davison et al. (2002) showed that girls who are practicing aesthetic sports report more concern about their weight and body shape than those practicing non-aesthetic sports. In addition, it is well known that extrinsic motives—Appearance included—are not as effective as intrinsic motives in promoting PA adherence (Ryan et al., 1997).

Finally, regarding Fitness/Health motives, Handball had lower scores than several other PAs. In line with this, Handball participants showed, in contrast, high levels of intrinsic and social motivation. This supports a previous study in which handball players showed high levels of basic psychological needs (Alesi et al., 2019), thus reflecting benefits for their mental health through self-determination fulfillment (Ryan and Deci, 2000). In other words, in our sample, Handball and other sports participants may focus on participating for motives such as interest/enjoyment, competence, and social, acquiring health benefits without explicitly (or strongly) seeking them.

Despite focusing on a less studied Latin American population, our study has several limitations. Among them, our sampling was non-randomized (i.e., by convenience), possibly reducing generalization and external validation. Our sample was also predominantly composed of university students. Thus, more diverse and representative samples are needed in future studies. In addition, our design was cross-sectional, and a longitudinal observation would offer complementary explanative power. Also, qualitative research would be welcomed, possibly exploring more possibilities for grouping PA according to unique interactions capable of improving understanding of the phenomenon. For example, Barreira (2018) proposed different subcategories for martial arts through phenomenological reduction. The same could be done in other PAs, improving understanding and opening up new avenues for further interventions. Together with more quantitative research, such as shown in the present study, a mixed qualitative-quantitative method, long-term observational studies, and studies in different socio-cultural settings would be highly beneficial for improving the area of motivation toward PAs.

## Conclusion

Our study shows that we can clearly distinguish several PAs and group them based on motivational subscales. For example, grouping some of them as team sports (Handball, Soccer, Basketball, Volleyball, and Rugby) is justified by their similar scores in Social motivation. More specifically, we showed that one set of PA groups (those classified as more Complex, Team, Vigorous, Hybrid, and Combative) scored higher on intrinsic motivation, while extrinsic motivations of Appearance and Fitness/Health identified the other set of PA groups (characterized as more Organized, Individual, Moderate, Strength, and Rhythmic). We thus reinforce the persistence of similar patterns in the distribution of PA regarding motivational subscales, despite using different criteria to classify PA. Using motivation as a grouping factor, our results provided initial evidence toward possible new methods of grouping PA types that can improve maintenance behavior. A more precise classification based on underlying motivations may contribute to improvements in health promotion by increasing individuals’ chances of choosing a PA that is more suitable to their unique psychological needs. Individualized PA may, in turn, enable more pleasurable sensations and, consequently, higher maintenance. For example, a personalized training program could be developed based on psychometric questionnaires analyzing individual psychological needs. Based on a dataset from previous studies, the training program could suggest some PA that is more matched with the person’s psychological scores. Possible changes could be measured periodically, as usually happens with other physiological variables, such as VO2, heart rate, rate of perceived exertion, etc. See also Hsu (2022) for additional and more detailed discussions regarding this topic.

Finally, our results show similar patterns of distribution of motives among different PA categories, suggesting a psychological-oriented corroboration. Future studies with different samples may better dissect similarities and differences among the classification systems in order to propose a stronger instrument to classify PAs according to different motives and possibly provide theoretical advancements.

Creating a motivational compendium of PAs could help to reduce drop-out and improve the personalization of interventions by using other measures such as each person’s personality, gender cognition, gender identity, sexual orientation, expectations, and idealized motives to find a better match for them. We hope that our findings and conclusions can inspire programs aimed at increasing adherence to PA in order to reduce many widespread sedentary-related health conditions and risk factors.

## Data Availability Statement

As additional analyses are still ongoing, the datasets presented in this article are not publicly available, and will be made available after the analyses are completed. Requests to access the datasets should be directed to RSH, rafa.mcsh@gmail.com.

## Ethics Statement

The project was approved by the IRB of the Institute of Psychology, University of São Paulo (number 1.506.899, approved on 19 April 2016). The patients/participants provided their written informed consent to participate in this study.

## Author Contributions

RSH, JVV, and FC contributed to the conception and design of the study, and performed the statistical analysis. RSH organized the database and wrote the first draft of the manuscript. All authors contributed to all sections of the manuscript and contributed to manuscript revision, read, and approved the submitted versions.

## Conflict of Interest

The authors declare that the research was conducted in the absence of any commercial or financial relationships that could be construed as a potential conflict of interest.

## Publisher’s Note

All claims expressed in this article are solely those of the authors and do not necessarily represent those of their affiliated organizations, or those of the publisher, the editors and the reviewers. Any product that may be evaluated in this article, or claim that may be made by its manufacturer, is not guaranteed or endorsed by the publisher.

## References

[B1] Ainsworth B. E. Haskell W. L. Herrmann S. D. Meckes N. Bassett D. R. Jr. Tudor-Locke C. (2011). Compendium of Physical Activities: a second update of codes and MET values. *Med. Sci. Sports Exerc.* 43 1575–1581. 10.1249/MSS.0b013e31821ece12 21681120

[B2] Alesi M. Gómez-López M. Chicau Borrego C. Monteiro D. Granero-Gallegos A. (2019). Effects of a motivational climate on psychological needs satisfaction, motivation and commitment in teen handball players. *Int. J. Environ. Res. Public Health* 16:2702. 10.3390/ijerph16152702 31362380 PMC6696366

[B3] Andrade Bastos A. Salguero A. González-Boto R. Marquez S. (2006). Motives for participation in physical activity by Brazilian adults. *Percept. Motor Skills* 102 358–367. 10.2466/pms.102.2.358-367 16826657

[B4] Apostolou M. Lambrianou R. (2017). What Motivates People to Do and Watch Sports? Exploring the Effect of Sex, Age, Partner Status, and Parenthood. *Evol. Psychol. Sci.* 3 20–33. 10.1007/s40806-016-0071-7

[B5] Baumeister R. F. (2016). Toward a general theory of motivation: problems, challenges, opportunities, and the big picture. *Motivat. Emot.* 40 1–10. 10.1007/s11031-015-9521-y

[B6] Barreira C. R. A. (2018). “Essência da luta e artes marciais,” in *Proceedings of the III Congresso da Associação Latina de Filosofia do Esporte*, São Paulo.

[B7] Baumeister R. F. Leary M. R. (1995). The need to belong: desire for interpersonal attachments as a fundamental human motivation. *Psychol. Bull.* 117, 497–529. 10.1037/0033-2909.117.3.497 7777651

[B8] Borges J. C. de Oliveira Filho G. G. de Lira C. A. B. da Silva R. A. D. Alves E. D. S. Benvenutti M. J. (2021). Motivation Levels and Goals for the Practice of Physical Exercise in Five Different Modalities: A Correspondence Analysis. *Front. Psychol.* 12:793238. 10.3389/fpsyg.2021.793238 34992570 PMC8724760

[B9] Cardoso F. L. Medeiros T. E. Da Silva W. R. Ferrari E. P. Melo G. F. (2020). A vivência de práticas físicas/motoras/esportivas de homens e mulheres para propor o construto orientação esportiva. *Revista Brasileira de Educação Física e Esporte* 34 395–404. 10.11606/1807-5509202000030395

[B10] Caspersen C. J. Powell K. E. Christenson G. M. (1985). Physical activity, exercise, and physical fitness: definitions and distinctions for health-related research. *Public Health Rep.* 100 126–131. 3920711 PMC1424733

[B11] Davis A. C. Arnocky S. (2022). An evolutionary perspective on appearance enhancement behavior. *Arch. Sex. Behav.* 51 3–37. 10.1007/s10508-020-01745-4 33025291

[B12] Davison K. K. Earnest M. B. Birch L. L. (2002). Participation in aesthetic sports and girls’ weight concerns at ages 5 and 7 years. *Int. J. Eat. Disorder.* 31 312–317. 10.1002/eat.10043 11920993 PMC2530926

[B13] Deaner R. O. Balish S. M. Lombardo M. P. (2015). Sex differences in sports interest and motivation: an evolutionary perspective. *Evol. Behav. Sci.* 10 73–97. 10.1037/ebs0000049

[B14] Duranso C. W. (2019). Walk for well-being: the main effects of walking on approach motivation. *Motivat. Emotion* 43 93–102. 10.1007/S11031-018-9726-Y

[B15] Frederick C. Ryan R. M. (1993). Differences in motivation for sport and exercise and their relations with participation and mental health. *J. Sport Behav.* 16 124–146.

[B16] Hsu R. M. C. S. (2022). *Motives for Practice of Physical Activities: An Evolutionary Approach.* (Ph.D.thesis). São Paulo, SP: University of São Paulo.

[B17] Hsu R. M. C. S. Valentova J. V. (2020). Motivation for different physical activities: a comparison among sports, exercises and body practices. *Psicol. USP* 31 1–10. 10.1590/0103-6564e190153

[B18] Jagacinski C. M. Strickland O. J. (2000). Task and ego orientation: the role of goal orientations in anticipated affective reactions to achievement outcomes. *Learn. Individ. Differ.* 12 189–208. 10.1016/S1041-6080(01)00037-1

[B19] Jenkins M. Houge Mackenzie S. Hodge K. Hargreaves E. A. Calverley J. R. Lee C. (2021). Physical Activity and Psychological Well-Being During the COVID-19 Lockdown: Relationships With Motivational Quality and Nature Contexts. *Front. Sports Active Living* 3:637576. 10.3389/fspor.2021.637576 33733237 PMC7959839

[B20] Kenrick D. T. Griskevicius V. Neuberg S. L. Schaller M. (2010). Renovating the pyramid of needs: contemporary extensions built upon ancient foundations. *Perspect. Psychol. Sci.* 5 292–314. 10.1177/1745691610369469 21874133 PMC3161123

[B21] Lee H. R. (2018). *An Investigation of the Pedagogic and Motivational Climate in UK Based Pilates Classes.* (Ph.D.thesis). Gloucestershire: University of Gloucestershire.

[B22] Lidborg L. H. Cross C. P. Boothroyd L. G. (2022). A meta-analysis of the association between male dimorphism and fitness outcomes in humans. *Elife* 11:e65031. 10.7554/eLife.65031 35179485 PMC9106334

[B23] Memmedova K. (2018). Quantitative analysis of effect of Pilates exercises on psychological variables and academic achievement using fuzzy logic. *Qual. Quant.* 52 195–204. 10.1007/s11135-017-0601-9

[B24] Molanorouzi K. Khoo S. Morris T. (2015). Motives for adult participation in physical activity: type of activity, age, and gender. *BMC Public Health* 15:66. 10.1186/s12889-015-1429-7 25637384 PMC4314738

[B25] Morris J. N. Hardman A. E. (1997). Walking to health. *Sports Med.* 23 306–332. 9181668 10.2165/00007256-199723050-00004

[B26] Murray S. B. Griffiths S. Mond J. M. Kean J. Blashill A. J. (2016). Anabolic steroid use and body image psychopathology in men: delineating between appearance-versus performance-driven motivations. *Drug Alcohol Depend.* 165 198–202. 10.1016/j.drugalcdep.2016.06.008 27364377

[B27] Naylor J. C. Briggs G. E. (1963). Effects of task complexity and task organization on the relative efficiency of part and whole training methods. *J. Experiment. Psychol.* 65 217–224. 10.1037/h0041060 13937802

[B28] Niven A. G. Markland D. (2016). Using self-determination theory to understand motivation for walking: instrument development and model testing using Bayesian structural equation modelling. *Psychol. Sport Exerc.* 23 90–100. 10.1016/j.psychsport.2015.11.004

[B29] Reeve J. (2018). *Understanding Motivation and Emotion.* New Jersey, US: John Wiley & Sons.

[B30] Roberts G. C. Nerstad C. G. (2020). “Achievement goal theory in sport and physical activity,” in *The Routledge International Encyclopedia of Sport and Exercise Psychology: Volume 1: Theoretical and Methodological Concepts*, eds Hackfort D. Schinke R. J. (London: Routledge), 322–341.

[B31] Ryan R. Frederick C. Lepes D. Rubio N. Sheldon K. (1997). Intrinsic motivation and exercise adherence. *Int. J. Sport Psychol.* 28 335–354.

[B32] Ryan R. M. Deci E. L. (2000). Self-determination theory and the facilitation of intrinsic motivation, social development, and well-being. *Am. Psychol.* 55 68–78. 10.1037//0003-066x.55.1.68 11392867

[B33] Shizgal P. (2001). “Motivation,” in *The MIT Encyclopedia of the Cognitive Sciences*, eds Wilson R. Keil F. (Cambridge, MA: MIT Press), 566–568.

[B34] Streb A. R. Matias T. S. Leonel L. D. S. Tozetto W. R. Vieira C. G. Del Duca G. F. (2019). Association between physical inactivity in leisure, work, commuting, and household domains and nutritional status in adults in the capital cities of Brazil. *Revista de Nutrição* 32:e180276. 10.1590/1678-9865201932e180276

[B35] U.S. Department of Health and Human Services (1996). *Physical Activity and Health: A Report of the Surgeon General.* Atlanta, GA: U.S. Department of Health and Human Services, Centers for Disease Control and Prevention, National Center for Chronic Disease Prevention and Health Promotion.

[B36] Valentova J. V. Mafra A. L. Varella M. A. C. (2021). Enhancing the Evolutionary Science of Self-Presentation Modification. *Arch. Sex. Behav.* 51 79–84. 10.1007/s10508-021-01975-0 33738591

[B37] Varella M. A. C. (2021). Evolved Features of Artistic Motivation: Analyzing a Brazilian Database Spanning Three Decades. *Front. Psychol.* 12:769915. 10.3389/fpsyg.2021.769915 34992565 PMC8724029

[B38] Varella M. A. C. Valentova J. V. Fernández A. M. (2017). “Evolution of artistic and aesthetic propensities through female competitive ornamentation,” in *The Oxford Handbook of Female Competition*, ed. Fisher M. L. (New York, NY: Oxford University Press), 757–783.

