# Supplementary material for: Comparing Different Typologies of Physical Activities With a Focus on Motivation
Source: Front Psychol. 2022 May 13;13:790490. doi: 10.3389/fpsyg.2022.790490 (PMC9137393; doi:10.3389/fpsyg.2022.790490)
Supplement: Supplementary file 1 [file Data_Sheet_1.docx]

Supplementary Material

# Supplementary Figures


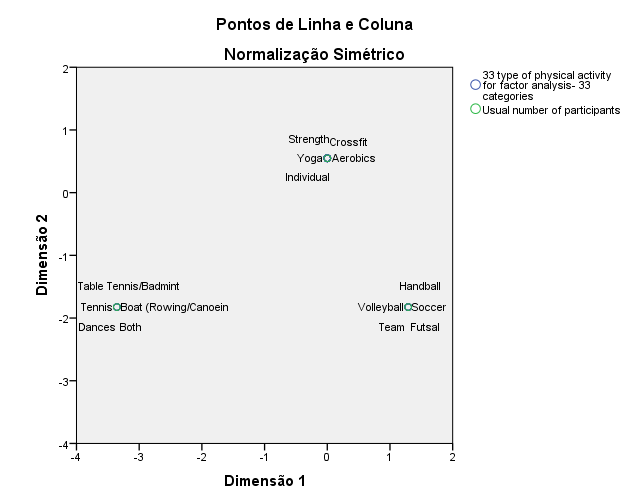


Supplementary Figure 1- Grouping of physical activities according to usual Number of Participants.


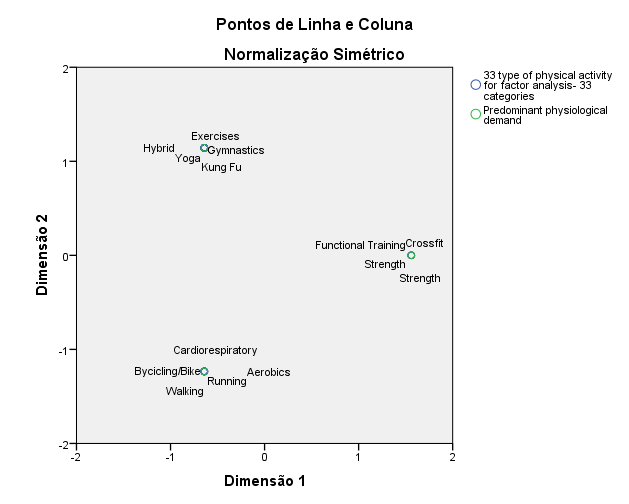


Supplementary Figure 2- Grouping of physical activities according to predominant physiological demand.


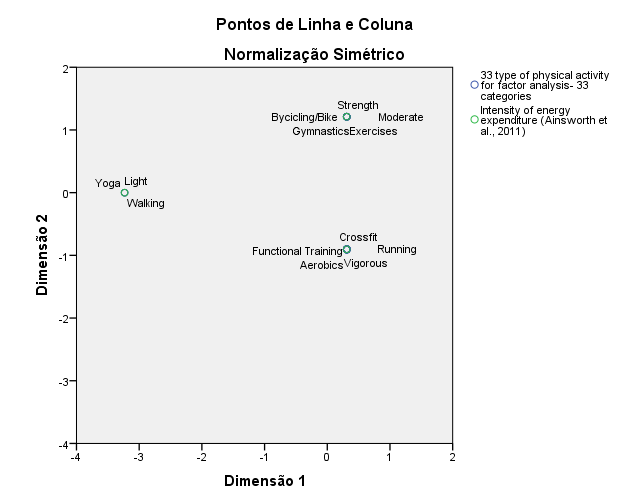


Supplementary Figure 3- Grouping of physical activities according to Intensity of Energy Expenditure.


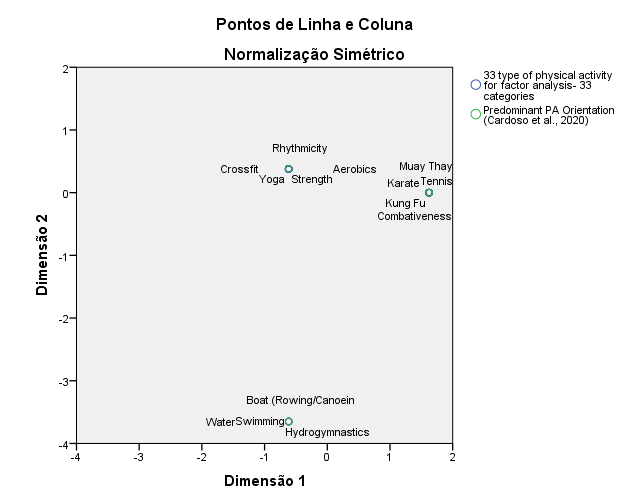


Supplementary Figure 4- Grouping of physical activities according to predominant Motor Orientation.
